# Changes in corneal aberrations and densitometry after diamond burr superficial keratectomy in post-traumatic recurrent corneal erosion: A retrospective observational study

**DOI:** 10.1097/MD.0000000000049212

**Published:** 2026-06-05

**Authors:** Han-Hao Tsai, Wei-Ting Ho

**Affiliations:** a Department of Ophthalmology, Far Eastern Memorial Hospital, New Taipei City, Taiwan; b School of Medicine, National Yang Ming Chiao Tung University, Hsinchu, Taiwan.

**Keywords:** corneal densitometry, diamond burr superficial keratectomy, high-order aberrations, recurrent corneal erosion

## Abstract

Recurrent corneal erosion (RCE) is a painful condition characterized by repeated breakdown of the corneal epithelium, often requiring interventions like diamond burr superficial keratectomy (DBSK) to promote healing. While DBSK is an effective treatment, its impact on corneal optical properties remains insufficiently studied. This study aims to assess changes in corneal aberrations and densitometry after DBSK in RCE patients. This retrospective case-control study enrolled patients with unilateral RCE treated with DBSK between January 2022 and September 2023 were reviewed. The fellow eye served as an internal control. Corneal low- and high-order aberrations (LOAs and HOAs) and densitometry were analyzed preoperatively, early postoperatively (≤2 weeks), and late postoperatively (2 weeks–3 months). Statistical analyses included paired t-tests and ANOVA. Ten eyes from ten patients (mean age: 32.4 years) were analyzed. Preoperatively, study eyes had higher LOAs (*P* = .047) but similar HOAs and densitometry compared to fellow eyes. Following DBSK, LOAs and HOAs were significantly reduced in the early and late postoperative period, separately. Among the individual components of LOAs and HOAs, defocus showed a significant reduction in the early postoperative phase. Anterior densitometry increased transiently but returned to baseline level by 3 months. DBSK improves corneal regularity by reducing aberrations while inducing only transient haze. These findings support its efficacy and safety for RCE management.

## 1. Introduction

Recurrent corneal erosion (RCE) is a clinical condition characterized by the repeated disruption of the corneal epithelium, leading to significant pain, visual disturbances, and decreased quality of life.^[1,2]^ The etiology of RCE is multifactorial, including prior ocular trauma, epithelial basement membrane dystrophy, stromal dystrophy, dry eye syndrome, diabetes mellitus, blepharitis, ocular rosacea, and previous ocular surgery, such as refractive surgery, cataract surgery and corneal transplantation.^[2–6]^ Most patients with RCE respond to conservative management, including topical antibiotics, artificial tears, hypertonic saline and lubricating ointments.^[5]^ For pain relief, cycloplegic eye drops or therapeutic soft bandage contact lens may be utilized, with oral analgesics provided as needed.^[5]^ Autologous serum eye drops may be considered as an alternative treatment option in more resistant cases.^[7]^ For patients with multiple recurrences, surgical intervention may become necessary.^[8]^

Surgical treatments for RCE include simple debridement, anterior stromal puncture, phototherapeutic keratectomy (PTK), and diamond burr superficial keratectomy (DBSK).^[5]^ Recurrence rates following these surgical interventions vary depending on the follow-up period. For anterior stromal puncture, the recurrence rate ranges from 17% to 37.1%.^[9,10]^ Following PTK, the recurrence rates vary between 0% and 36%.^[10–14]^ Among these treatments, DBSK is recognized as a highly effective and well-established technique, with recurrence rates reported between 4% and 11.1%.^[10,14–16]^ The procedure of DBSK involves the mechanical removal of loosened epithelium, thereby smoothing the corneal surface to promote epithelial adhesion during the wound healing process.^[16]^ Previous studies showed that DBSK is a generally safe procedure without inducing significant complications.^[16]^ However, some reports suggested that it can cause additional astigmatism and corneal haze, raising concerns about its impact on visual quality.^[16–20]^ Furthermore, given its potential to induce astigmatism, it is crucial to clarify whether DBSK also affects high-order aberrations (HOAs) and corneal clarity.

In this study, we aim to analyze the serial changes of low and high order corneal aberrations (LOAs and HOAs) as well as corneal densitometry to determine the impact of DBSK on the recovery of corneal contour and clarity in RCE patients.

## 2. Methods

### 2.1. Study design

This study was a retrospective case-control study adhered to the Declaration of Helsinki and was approved by the Institutional Review Board of Far Eastern Memorial Hospital, New Taipei City, Taiwan (IRB #111164-E). Medical records of patients with RCE undergoing DBSK from January 2022 to September 2023 were reviewed. The inclusion criteria were RCE caused by trauma in single eye, so the fellow eye can serve as the internal control. Exclusion criteria were RCE attributed to other etiologies, such as epithelial basement membrane dystrophy, stromal corneal dystrophies, herpetic keratitis, bullous keratopathy, or band keratopathy.

Demographic data and clinical examination data of the enrolled patients were collected, including age, sex, best corrected visual acuity (BCVA), intraocular pressure (IOP, measured by iCare rebound tonometer), refractive spherical equivalent, refractive astigmatism, and data from Scheimpflug imaging examination (Pentacam HR, Oculus, Wetzlar, Germany). Clinical data were collected in 3 phases: 1. preoperative baseline data were reviewed from records prior to the DBSK procedure; 2. early postoperative data were collected from records within the first 2 weeks following the procedure; 3. late postoperative data were obtained from records covering the period between 2 weeks and 3 months post-DBSK. The parameters from Scheimpflug imaging examination gathered in this study included low-order aberrations (LOAs, including defocus and astigmatism), HOAs (including coma, trefoil, spherical aberration) and total aberrations, which were expressed as root-mean-square (root-mean-square [RMS], µm) units. Additionally, since DBSK may cause corneal haze, corneal densitometry data measured in grayscale units (GSU) ranging from 0 (maximum transparency) to 100 (minimum transparency) were obtained from Scheimpflug imaging. The data included measurements from the anterior layer (the outermost 120 μm), the posterior layer (the innermost 60 μm), and the middle layer. The corneal densitometry map from Scheimpflug imaging was subdivided into 4 concentric radial zones centered on the apex: the central 0 to 2 mm zone, the 2 to 6 mm annulus, the 6 to 10 mm annulus, and the outermost 10 to 12 mm annulus. Since DBSK was performed within the 10 mm corneal zone, the average GSU within this zone from the 3 corneal layers were used for further analysis.

### 2.2. Surgical procedure

To perform DBSK, topical anesthesia was administered by instilling Proparacaine® Oph. Solution 0.5% (WU FU LABORATORIES CO., LTD., Yilan, Taiwan). After placing eyelid speculum, the loosened corneal epithelium was initially removed using 0.12-mm forceps. Subsequently, a hand-held, air-driven diamond burr (4.0 mm diameter, Medtronic Xomed Surgical Products, Inc., Jacksonville) was used to gently and uniformly polish the Bowman membrane over the entire area of the epithelial defect for approximately 10 seconds. Postoperatively, a bandage soft contact lens was applied and topical 0.3% norfloxacin eye drops 4 times daily were administered for 1 week. After full epithelialization, preservative-free lubricant was administered.

### 2.3. Statistical analysis

The data were analyzed using GraphPad Prism version 9.0.0 for Windows (GraphPad Software, Boston, MA, USA) The normality of variables was tested with the Shapiro–Wilk test. Parametric datasets were analyzed using the paired t-test, while nonparametric datasets were analyzed using Wilcoxon signed-rank test. Results from the 3 distinct phases were compared using 1-way repeated measures ANOVA with a Tukey post hoc test. A *P*-value < 0.05 was considered statistically significant.

## 3. Results

A total of 10 eyes from 10 patients (4 males and 6 females), aged 22 to 46 years with a mean age of 32.4 years, met the inclusion criteria and were analyzed. The preoperative characteristics of study eyes and fellow eyes were summarized in Table 1. No significant differences were observed between study eyes and fellow eyes in BCVA, IOP, refractive spherical equivalent, refractive astigmatism, topographic astigmatism, or densitometry. However, LOAs were significantly higher in study eyes (2.67 ± 1.35 µm vs 1.8 ± 0.78 µm, *P* = .047), while total aberrations were marginally higher (2.77 ± 1.36 µm vs 1.95 ± 0.66 µm, *P* = .056). Before DBSK treatment, there were no differences between study eyes and fellow eyes in HOAs (0.74 ± 0.24 µm vs 0.61 ± 0.26 µm, *P* = .272), total densitometry (14.6 ± 1.1 GSU vs 14.2 ± 2.1 GSU, *P* = .313) and anterior densitometry (19.5 ± 1.9 GSU vs 18.7 ± 3.2 GSU, *P *= .272).

**Table 1:** Preoperative demographics.

Characteristics	Preoperative		
	Study eyes	Fellow eyes	
	(n = 10)	(n = 10)	*P*-value
Best corrected visual acuity (LogMAR)	0.1 ± 0.14	0.08 ± 0.25	–
Intraocular pressure (mm Hg)	16.1 ± 4.3	17.3 ± 3.1	.397
Refraction spherical equivalent (D)	−1.95 ± 1.79	−1.98 ± 2.34	.943
Autorefractive astigmatism (D)	−1.5 ± 1.42	−0.63 ± 0.34	.051
Topographic astigmatism (D)	−1.16 ± 1.19	−0.89 ± 0.84	.127
Topographic aberration (RMS, µm)			
Total aberrations	2.77 ± 1.36	1.95 ± 0.66	.056
High order aberrations	0.74 ± 0.24	0.61 ± 0.26	.272
Low order aberrations	2.67 ± 1.35	1.8 ± 0.78	.047
Densitometry (GSU)			
Total	14.6 ± 1.1	14.2 ± 2.1	.313
Anterior	19.5 ± 1.9	18.7 ± 3.2	.272
Center	12.7 ± 1.0	12.4 ± 1.9	.404
Posterior	11.6 ± 0.8	11.5 ± 1.3	.477

*P*-values were calculated using the paired *t*-test.

D = diopters, GSU = grayscale units, LogMAR = logarithm of the minimum angle of resolution, RMS = root-mean-square.

**Figure 1.**
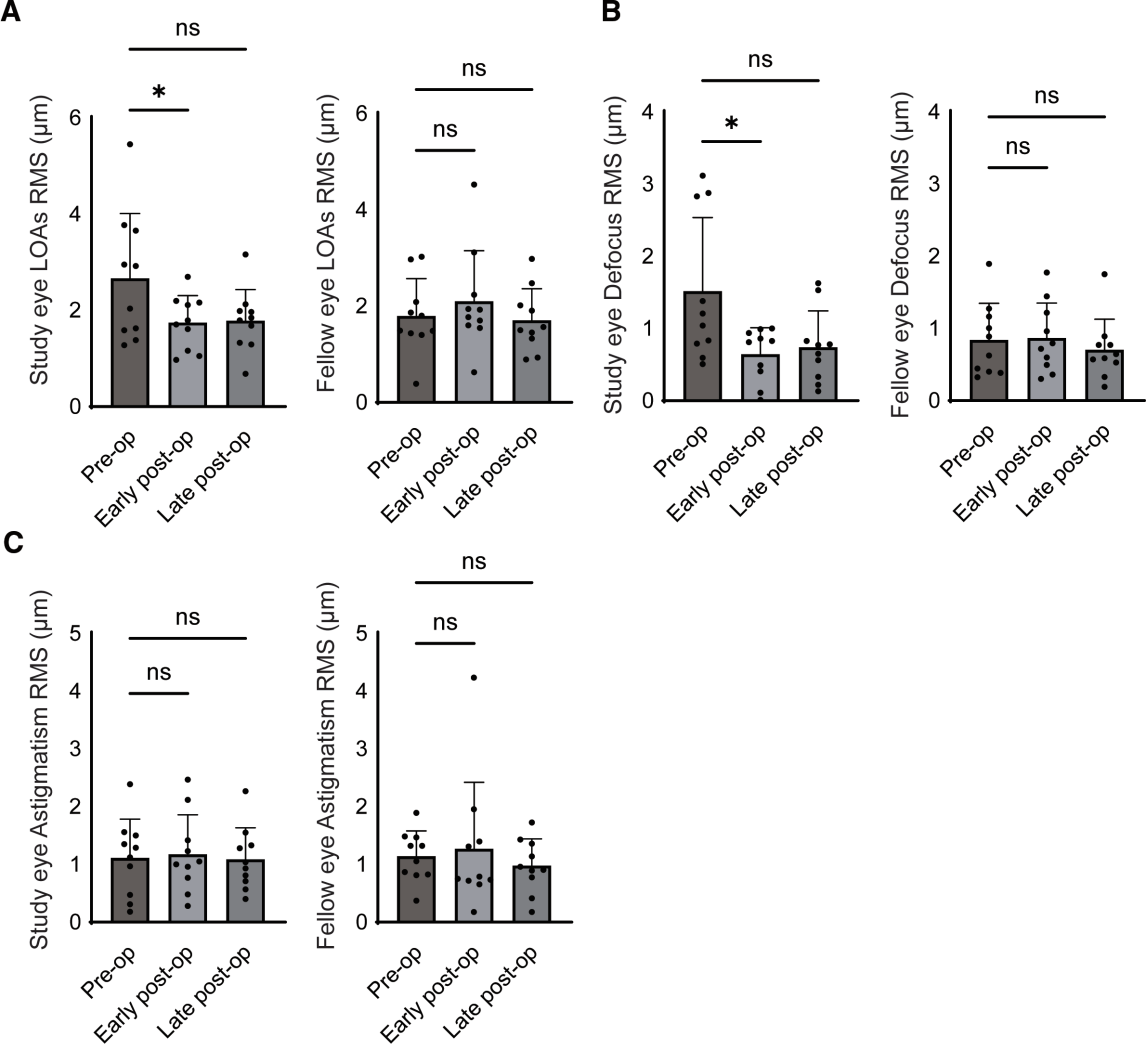
Total (A) and individual low order aberrations, including defocus (B) and astigmatism (C) in study eyes (left panel) and fellow eyes (right panel) before and after DBSK. Data are shown as mean ± SD. *P < .05, ns by analysis of variance with Tukey post hoc test. DBSK = diamond burr superficial keratectomy, ns = non-significant, RMS = root mean square.

**Figure 2.**
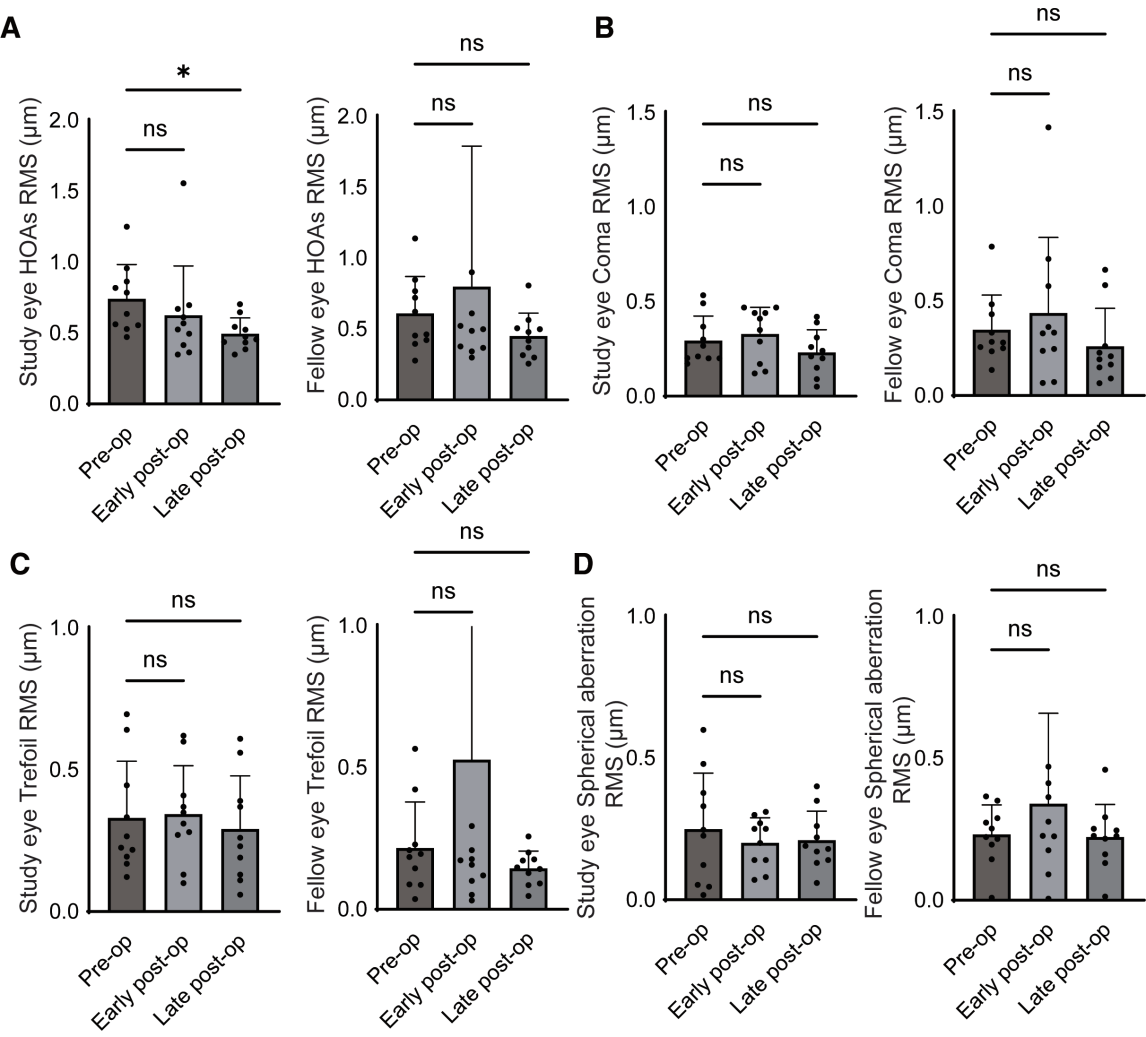
Total (A) and individualHOAs, including coma (B), trefoil (C) and spherical aberration (D) in study eyes (left panel) and fellow eyes (right panel) before and after DBSK. An outlier with a value of 3.967 in the early postoperative group in the right panel of figure (D) was not shown. Data are shown as mean ± SD. *P < .05, ns by analysis of variance with Tukey post hoc test. DBSK = diamond burr superficial keratectomy, HOAs = high-order aberrations, ns = non-significant, RMS = root mean square.

**Figure 3.**
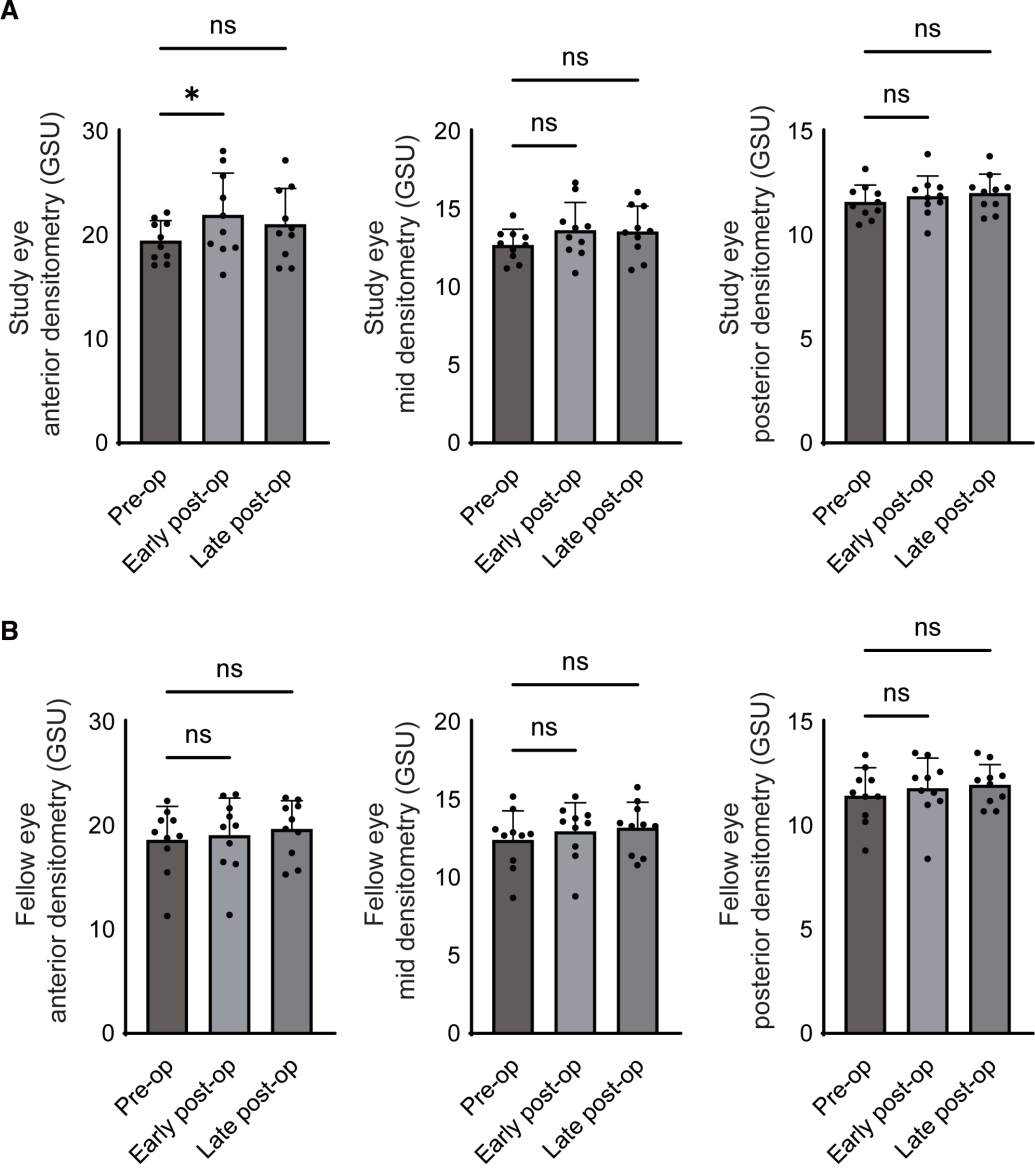
Densitometry of anterior, mid and posterior corneal layers in study eyes (A) and fellow eyes (B) before and after DBSK. Data are shown as mean ± SD. *P < .05, ns by analysis of variance with Tukey post hoc test. DBSK = diamond burr superficial keratectomy, ns = non-significant.

After DBSK treatment, BCVA in study eyes showed a minimal improvement from 0.1 ± 0.14 logMAR to 0.04 ± 0.1 logMAR in the early postoperative period, and to 0.03 ± 0.05 logMAR in the late postoperative period, which became comparable to the fellow eyes (0.08 ± 0.25 logMAR). Astigmatism measured by autorefractor also improved, decreasing from − 1.50 ± 1.42 D preoperatively to − 0.61 ± 0.28 D in the early postoperative period and to − 0.53 ± 0.46 D in the late postoperative period, approaching the levels observed in fellow eyes (−0.58 ± 0.33 D). Total LOAs in the study eyes were significantly reduced in the early postoperative period, while those in the fellow eyes remained unchanged (Fig. 1A). Meanwhile, total HOAs in the study eyes were marginally lowered in the early postoperative period, and were further reduced in the late postoperative period (Fig. 2A). Next we performed further analysis of individual components of LOAs and HOAs. Among LOAs, defocus RMS was significantly reduced in the early postoperative period compared with the preoperative values (Fig. 1B). However, the astigmatism component of LOAs as well as the individual components of HOAs, including coma, trefoil, and spherical aberration, did not significantly differ before and after the surgery (Figs. 1C, 2B–D). We further analyzed the change in corneal densitometry after DBSK, for previous studies showed that the procedure may induce corneal haze.^16–20^ In early postoperative period, DBSK treatment led to significant increase in anterior densitometry in the study eyes, which returned to baseline levels in the late postoperative period (Fig. 3). On the contrary, the anterior densitometry in the fellow eyes, as well as the densitometry in the middle or posterior corneal layer of both eyes, remained unaffected after DBSK (Fig. 3).

**Figure 4.**
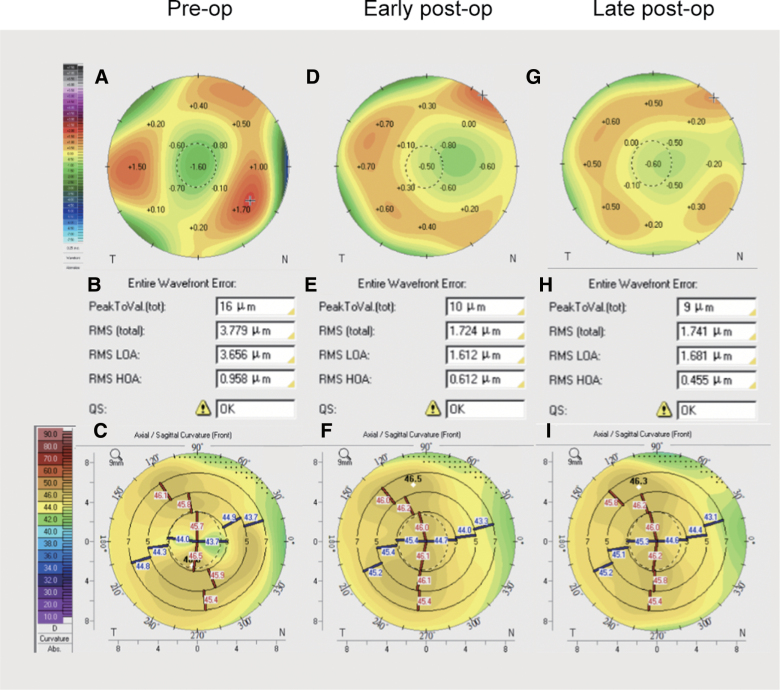
Representative aberrometry map before and after DBSK. DBSK = diamond burr superficial keratectomy.

**Figure 5.**
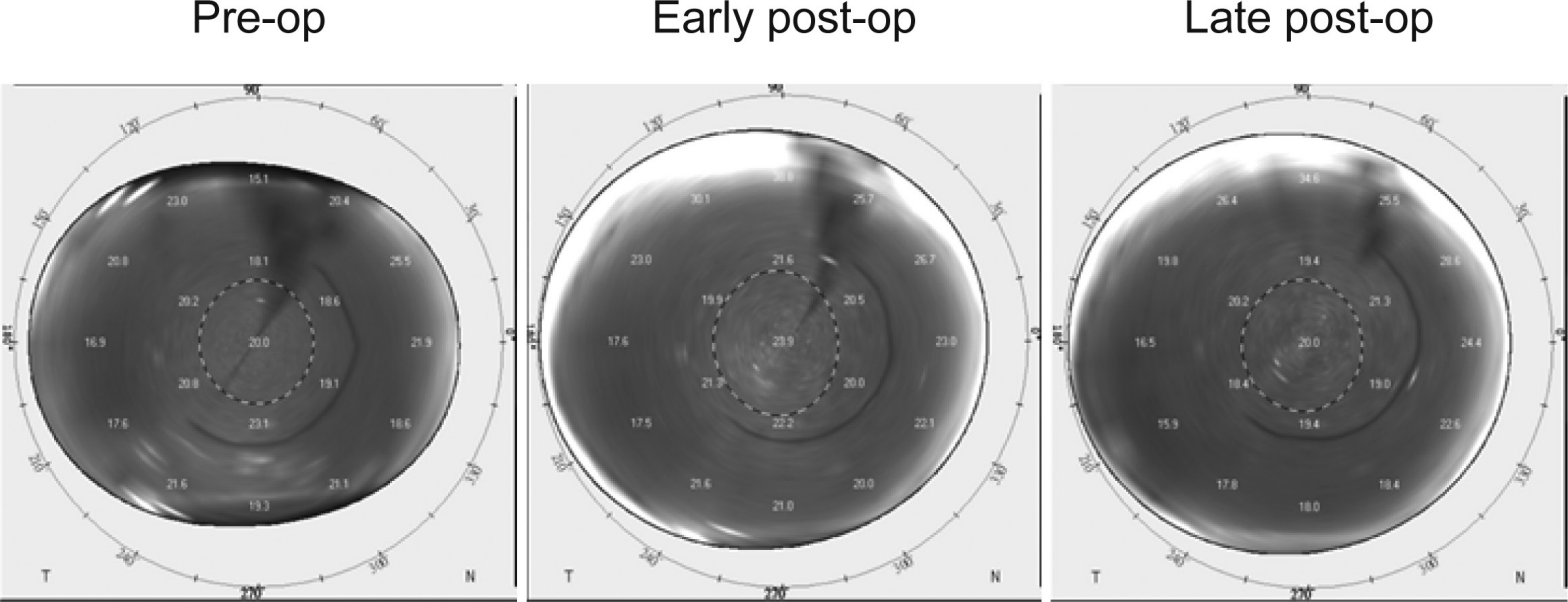
Representative anterior corneal densitometry map before and after DBSK. DBSK = diamond burr superficial keratectomy.

Figure 4 demonstrated the changes in wavefront aberrometry analysis following DBSK in a representative case of RCE. Preoperatively, both LOAs and HOAs were elevated, indicating significant corneal surface irregularity. Postoperatively, both LOAs and HOAs exhibited a marked decrease, suggesting improved corneal smoothness and optical quality. In another representative case, corneal densitometry analysis demonstrated a transient increase in densitometry in early postoperative period, indicating reactive haze. However, by the late postoperative phase, densitometry values returned to baseline, indicating resolution of haze and restoration of corneal transparency (Fig. 5). During the follow-up period, no patients experienced recurrence of corneal erosion. These findings demonstrated the temporary nature of post-surgical corneal haze and the long-term safety of DBSK in treating RCE patients.

## 4. Discussion

Our study demonstrated that DBSK effectively reduced both LOAs and HOAs in patients with RCE. Additionally, while DBSK induced a transient increase in corneal densitometry in the early postoperative period, this effect resolved over time, returning to baseline levels in the late postoperative period. These findings suggest that DBSK not only promotes epithelial adhesion and corneal surface regularity but also maintains long-term corneal clarity, supporting its efficacy and safety as a treatment for RCE.

The reduction in LOAs and HOAs observed in this study aligns with previous reports indicating that DBSK can improve corneal smoothness and optical quality.^[15,16,18]^ Our analysis showed that LOAs were elevated in study eyes compared with fellow eyes before surgery and were significantly reduced after DBSK, particularly defocus RMS. LOAs, mainly composed of defocus and astigmatism, account for nearly 90% of monochromatic aberrations and are strongly influenced by gross corneal surface irregularity.^[21]^ In RCE, repeated epithelial damage and irregular healing create surface irregularities that alter the effective corneal curvature, particularly in the affected regions.^[5]^ By mechanically smoothing the anterior surface, DBSK effectively reduces these irregularities, leading to improvement in LOAs. In contrast, although total HOA RMS decreased following DBSK, the individual components, including coma, trefoil, and spherical aberration, did not change significantly. This discrepancy may reflect a global smoothing effect of DBSK that produces modest reductions across multiple HOAs, which cumulatively lower total HOAs RMS but fail to reach statistical significance in each component. Moreover, HOAs constitute a smaller proportion of total aberrations and are influenced by factors beyond corneal surface morphology, such as tear film instability. The relatively small sample size in the present study may further obscure subtle changes in individual HOAs. Larger studies are warranted to clarify the contribution of specific HOAs to visual quality in RCE and to better define the effects of DBSK on HOAs.

One of the major concerns regarding DBSK is the potential development of corneal haze or induced astigmatism, which could affect visual outcomes.^[17,19,20]^ Our findings revealed a transient increase in anterior corneal densitometry in the early postoperative phase, indicating temporary corneal haze. However, by the late postoperative period, densitometry values returned to preoperative levels, suggesting that the haze was reversible. The transient nature of the corneal haze may be due to the wound healing process, as keratocyte activation and extracellular matrix deposition initially contribute to increased corneal scattering, which later subsides with full epithelial and basement membrane restoration.^[22]^ In previous studies using DBSK to treat other corneal pathologies, such as RCE and visually significant irregularity associated with anterior basement membrane dystrophy, a subset of eyes develop mild subepithelial haze, typically without a measurable reduction in best-corrected visual acuity.^[14,18,23]^ Our study extends these observations by focusing specifically on traumatic RCE and by quantifying the time course of haze using Scheimpflug-based corneal densitometry. In addition, we found that the transient increase in corneal densitometry did not interfere with visual acuity even without the use of topical steroids. Nevertheless, short-term administration of topical steroids could be considered as a reasonable option to further minimize the risk of haze formation and enhance the safety profile of DBSK in the management of RCE.

A key strength of this study is the use of the fellow eye as an internal control, allowing for a more precise comparison of postoperative changes in the treated eye. The consistency between preoperative baseline characteristics in study and fellow eyes further supports the reliability of our findings. However, this study has several limitations. First, the sample size was relatively small, which may limit the generalizability of our results. Second, the follow-up period was limited to 3 months, and longer-term studies are needed to assess whether corneal aberrations and densitometry remain stable beyond this period. Lastly, although Scheimpflug imaging provides objective measurements of corneal aberrations and densitometry, additional functional visual assessments, such as contrast sensitivity testing or patient-reported outcome measures, could provide further insight into the clinical impact of DBSK.

In conclusion, DBSK effectively reduces LOAs and HOAs in patients with RCE, leading to improved corneal regularity and visual quality. Although the procedure induces transient corneal haze, this effect resolves over time, indicating that DBSK is a safe and effective surgical option for RCE. Future studies with larger cohorts and extended follow-up periods are warranted to further evaluate the long-term outcomes of this procedure.

## Author contributions

**Conceptualization:** Wei-Ting Ho.

**Data curation:** Han-Hao Tsai, Wei-Ting Ho.

**Investigation:** Wei-Ting Ho.

**Writing – original draft:** Han-Hao Tsai.

**Writing – review & editing:** Wei-Ting Ho.
